# Amino-Functionalized Silica@Resorcinol–Formaldehyde Nanocomposites for the Removal of Cr(VI) from Aqueous Solutions

**DOI:** 10.3390/polym15204094

**Published:** 2023-10-15

**Authors:** Nan Li, Wenhui Lu, Deyi Zhu

**Affiliations:** 1 Faculty of Light Industry, Qilu University of Technology (Shandong Academy of Sciences), Jinan 250353, China; zhdy@qlu.edu.cn; 2 Key Laboratory for Green Technology of Leather Manufacture, China National Light Industry Council, Jinan 250353, China

**Keywords:** Cr(VI), silica@resorcinol-formaldehyde, nanocomposite, adsorption, aqueous solution

## Abstract

Amino-functionalized silica@resorcinol–formaldehyde nanocomposites (NH_2_-SiO_2_@RF) were synthesized for the removal of Cr(VI) from aqueous solutions using the sol–gel technique with two simple preparation steps, including the one-pot synthesis of SiO_2_@RF using the Stöber method and (3-aminopropyl)triethoxysilane (APTES) modification. The morphology, particle size, functional group, and thermal stability of the obtained nanocomposites were systematically characterized, with the results indicating a uniform sphericity with a particle size of 200 nm and high thermal stability. The adsorption results demonstrated that the preferred pH value was 2, and the data were well fitted with the Langmuir and Temkin isotherm models and quasi-second-order kinetic equation, indicating a high adsorption capacity. The maximum Cr(VI) adsorption capacity from the nonlinear form of the Langmuir model was 272.6 mg·g^−1^. The intra-particle diffusion model accurately described the adsorption of Cr(VI) onto NH_2_-SiO_2_@RF. The changes in Gibb’s free energy, enthalpy, and entropy revealed that Cr(VI) adsorption onto NH_2_-SiO_2_@RF was a spontaneous and endothermic process. Furthermore, high selectivity was demonstrated in the material for the removal of Cr(VI) from commonly coexisting ions. The obtained nanocomposites had good regeneration properties and maintained a removal rate above 85% in the fifth adsorption–desorption experiments. Moreover, under the optimized adsorption conditions, the obtained nanocomposites were preliminarily applied to tannery wastewater, demonstrating an excellent removal effect, which indicates their potential application value.

## 1. Introduction

Hexavalent chromium (Cr(VI)) is widely used in industrial processes including tanning, electroplating, wood preserving, and pigmentation [1]. Cr(VI) is found not as free ions, but in a complex form in water, and all Cr(VI) species are highly soluble oxides (i.e., hydrochromate (HCrO_4_^−^), chromate (CrO_4_^2−^) and dichromate (Cr_2_O_7_^2−^) anions) [2]. Cr(VI) is highly toxic to plants, animals, and the human body and the Global Burden of Disease (GBD) study categorized Cr(VI) as an occupational carcinogen [3,4]. China has stipulated that the emission concentration of Cr(VI) in industrial wastewater discharges must not exceed 0.1 mg·L^−1^ [5]. Therefore, it is important to control the emission of Cr(VI).

Currently, various technologies, including chemical treatment [6], membrane separation [7], biodegradation [8], electrochemical technology [9], and adsorption [10] are used for processing Cr(VI) in sewage. Among these technologies, the adsorption method is simple, highly efficient, and has a good cost performance and low risk of secondary pollution, making it a very promising method [11]. For various adsorption methods, the adsorption effects are strongly affected by the type of adsorbent. Currently, the commonly used adsorbents mainly include activated carbon, zeolite, nanomaterials, biosorbents, etc. [12,13,14]. However, the weak sorption selectivity and low surface areas/site ratio obviously limits their practical utility. Organic–inorganic nanostructured composites with a high surface area are used in a variety of fields, especially for water treatment, as the supply of the adsorption sites and the skeleton are supported by the organic part and the inorganic part, respectively [11,15]. Amino-functionalized silica@resorcinol–formaldehyde nanocomposites (NH_2_-SiO_2_@RF) are a kind of organic–inorganic nanostructured composite, synthesized using the sol–gel technique with two simple preparation steps, including using the Stöber method for the one-pot synthesis of SiO_2_@RF, as well as the (3-aminopropyl) triethoxysilane (APTES) modification of SiO_2_@RF.

Silica spheres with a controllable narrow particle size distribution can be synthesized using alcohol–water solvents and an ammonia catalyst through hydrolysis and the condensation of silicon alkanes, which is known as the Stöber method [16]. Resorcinol–formaldehyde (RF) resin can also be synthesized using an ethanol–water–ammonia system [17]. From this, a SiO_2_@RF complex can be synthesized in the Stöber system through innovative methods such as prolonging the reaction time [18,19]. Nitrogen-containing functional groups such as aliphatic amines and aromatic amines can effectively enhance the adsorption effect of Cr(VI) [20,21]. The surface amine group tends to be positively charged, and such a tendency provides a good amine-positive Cr(VI) interaction. Therefore, amine-functionalized silica has attracted a great deal of attention [20,21,22,23]. For example, Jang et al. demonstrated the adsorption of Cr(VI) by amino-functionalized amorphous silica nanoparticles and mesoporous silica nanoparticles as well as the removal of Cr(VI) from water environments. The results suggested a structural and chemical rationale for enhanced Cr(VI) adsorption and removal. However, the adsorption capacity was not high, and there was a lack of practical applications for the adsorbent [21]. Granular N-(3-trimethoxysilylpropyl)diethylenetriamine-grafted mesoporous silica SBA-15 was synthesized, and the maximum Cr(VI) adsorption capacity was determined to be 330.9 mg·g^−1^ using the Langmuir model. To a certain extent, this overcame the limitations of powder adsorbents for large-scale applications (hydraulic and separation problems) [22]. However, the exact adsorption behavior of Cr(VI) on functionalized silica remains unclear, and many aspects have yet to be systematically studied.

Taking these factors into account, amino-functionalized silica@resorcinol–formaldehyde nanocomposites (NH_2_-SiO_2_@RF) were synthesized in two simple preparation steps, involving the silane reagent APTES-functionalized SiO_2_@RF. The nanocomposites were thoroughly characterized using scanning electron microscopy (SEM), a laser particle sizer, Fourier transform infrared (FT-IR) spectroscopy, and thermogravimetric analysis (TGA). In addition to the effects of the adsorbent dosage, solution pH, contact time, initial concentration, and temperature, its regeneration properties and coexistent ions were all systematically investigated. Both the nonlinearized and linearized forms of the Langmuir, Freundlich, Temkin, and Dubinin–Radushkevich (D-R) isotherm models were used to fit the test data and adsorption kinetic models (the pseudo-first-order model and pseudo-second-order model) were used to analyze the data. Finally, the selectivity and reusability of the materials were evaluated, and the prepared nanocomposites were used for the adsorption of actual tannery wastewater, showing a potential practical application value.

## 2. Experimental Procedure

### 2.1. Reagents and Materials

Tetraethoxysilane (TEOS), resorcinol, potassium dichromate (K_2_Cr_2_O_7_, ≥99.0%), 1,5-diphenyl carbazide (DPC), formaldehyde aqueous solution (37.0~40.0%), NH_3_·H_2_O (25.0~28.0%), absolute ethanol (EtOH), potassium chloride, cobalt chloride, manganese chloride, copper chloride, nickel chloride, lead nitrate, ferrous sulfate, chromium chloride, sodium carbonate, sodium sulfate, and sodium phosphate were all purchased from Sinopharm Chemical Reagent Co., Ltd. (Shanghai, China). 3-aminopropyltriethoxysilane (APTES) was purchased from Sigma-Aldrich (Shanghai, China). The other solvents and agents were analytically pure and used without further edulcoration. Deionized water was utilized throughout the whole experiment.

### 2.2. Preparation of Amino Functionalized Silica@resorcinol–Formaldehyde Nanocomposites (NH_2_-SiO_2_@RF)

**Figure 1:**
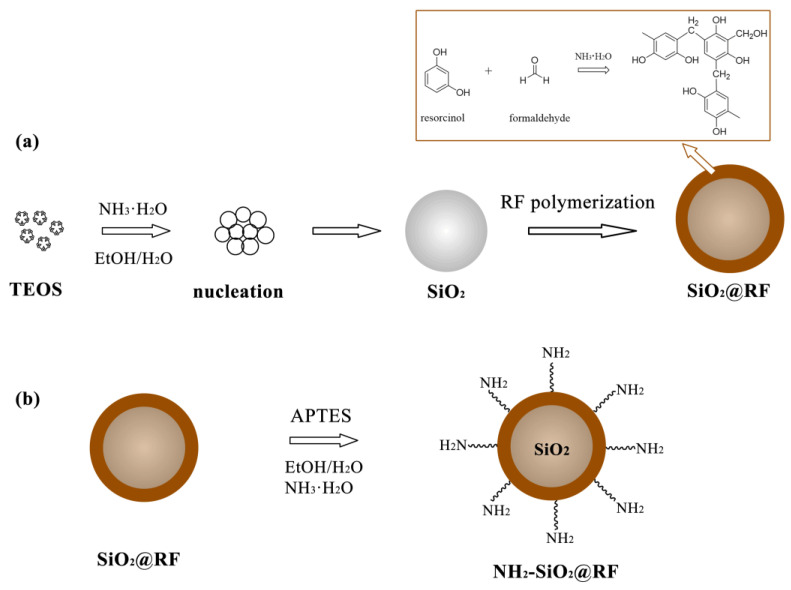
Preparation procedure of SiO_2_@RF (**a**) and NH_2_-SiO_2_@RF nanocomposites (**b**).

SiO_2_@RF nanocomposites were prepared using the one-step Stöber method, as schematically shown in Figure 1a. In brief, a 40 mL mixture of EtOH/H_2_O (3:1, volume) and 1.25 mL NH_3_·H_2_O were added into a three-necked flask with vigorous stirring for 30 min at 30 °C. Then, 1.4 mL TEOS, 0.25 g resorcinol, and 350 μL formaldehyde aqueous solution were added, in order, at 30 min intervals; stirring was carried out continuously for 24 h. Then, the mixture was centrifuged at 7500 rpm for 10 min. To remove the unreacted solvent, the mixture was washed several times using absolute ethanol and ultrapure water. Finally, the mixture was dried at 50 °C for 12 h. About 0.575 g of SiO_2_@RF was obtained after this step.

As shown in Figure 1b, the NH_2_-SiO_2_@RF nanocomposites were manufactured via the sol–gel method using APTES for the modification of SiO_2_@RF. In brief, 200 mg SiO_2_@RF was dispersed into a 90 mL mixture of EtOH/H_2_O (8:1, volume) via sonication, and 2 mL NH_3_·H_2_O was added into the solution while stirring at 30 °C. Then, 2 mL APTES was added dropwise. After 12 h of vigorous stirring, the mixture was centrifuged at 7500 rpm for 10 min. Then, the mixture was washed several times using absolute ethanol and ultrapure water. The final products were dried at 50 °C for 12 h. Finally, about 0.217 g NH_2_-SiO_2_@RF was obtained.

### 2.3. Characterization of NH_2_-SiO_2_@RF

The morphology of the NH_2_-SiO_2_@RF nanocomposites was characterized via scanning electron microscopy (SEM, Hitachi S-4800, Hitachi, Japan). The particle sizes were tested using a laser particle size meter (Nano ZS90, Malvern, UK). The specific surface area and porosity were measured using the Brunauer–Emmett–Teller (BET) and Barrett–Joyner–Halenda (BJH) methods (Bethdard, Beijing, China). The infrared spectra of the nanocomposites were examined using a Fourier transform infrared (FT-IR) spectrometer (Thermo Nicolet Corporation, Madison, WI, USA), with wavenumbers ranging from 400 to 4000 cm^−1^. Thermogravimetric analysis in a nitrogen atmosphere (Mettler 5MP/PF7548/MET/400W thermal analyzer, Mettler Toledo, Columbus, OH, USA) was used to assess the thermal stability of the nanocomposites from 40 to 700 °C with the condition of a nitrogen flow rate of 50 mL·min^−1^ and heating rate of 20 °C·min^−1^. 

### 2.4. Adsorption Studies

First, 1000 mg·L^−1^ of Cr(VI) stock solution was prepared by adding an appropriate amount of K_2_Cr_2_O_7_ to deionized water. Then, the desired concentrations of the Cr(VI) solution were prepared by dissolving the stock solutions. Batch adsorption studies were carried out by mixing varying dosages of NH_2_-SiO_2_@RF with 5 mL Cr(VI) solutions of varying concentrations in 10 mL round-bottomed centrifugal tubes. Then, the tubes were sealed and shaken in a vapor-bathing vibrator at 150 rpm. The various adsorption parameters, such as adsorbent dosages (0.3~4.0 g·L^−1^), pH value (1~9), concentrations (5~400 mg·L^−1^), and contact time (10~350 min), were varied independently to optimize the adsorption capacity. The pH value was regulated using HCl (1 mol·L^−1^) and NaOH (1 mol·L^−1^). After the above-mentioned adsorption procedures were carried out, the tubes containing the above-described mixture were centrifuged at 7500 rpm for 10 min. Then, the supernatant liquors were acidized using phosphoric acid and complexed with DPC, which was detected using a UV-vis spectrophotometer (Thermo Scientific NanoDrop 2000/2000C, Waltham, MA, USA) at the wavelength of 540 nm. All the adsorption experiments were performed three times, and the average value was taken for the final analysis.

The removal efficiency R (%) of Cr(VI) and the equilibrium adsorption capacity q_e_ (mg·g^−1^) were calculated using Equations (1) and (2),

(1) $$R=\frac{C_{0}-C_{e}}{C_{0}}\times \;100\%$$

(2) $$q_{e}=\frac{V(C_{0}-C_{e})}{m}$$

where C_0_ and C_e_ (mg·L^−1^) denote the initial concentration and adsorption equilibrium concentration of Cr(VI), respectively, V (mL) is the volume of the Cr(VI) solution, and m (mg) is the adsorbent mass used in the experiments.

### 2.5. Desorption Studies

To evaluate the reuse performance of NH_2_-SiO_2_@RF nanocomposites, adsorption–desorption experiments were carried out. BFirstly, 50 mg NH_2_-SiO_2_@RF nanocomposites was dispersed into 50 mL Cr(VI) with concentration of 30 mg·L^−1^ at pH 2.0, and the mixture was shaken for 4 h at 150 rpm and 25 °C. Then, the adsorbents were collected via centrifugation and rinsed with ultrapure water until reaching a neutral state. After being desorbed with 100 mL NaOH at a concentration of 0.5 mol·L^−1^, the adsorbents were collected and rinsed with ultrapure water until reaching a neutral state and dried in a vacuum oven at 50 °C for 12 h. Then, the regenerated NH_2_-SiO_2_@RF nanocomposites were reused in the next adsorption–desorption cycle.

## 3. Results and Discussion

### 3.1. Preparation and Characterization of NH_2_-SiO_2_@RF

In the process of synthesizing silica using the Stöber method, silica particles can be hydrolyzed by TEOS one hour after the reaction begins. The preparation of RF microspheres via the Stöber method under the same reaction conditions (e.g., reaction solvent, catalyst, temperature, etc.) takes longer (about 24 h). In this work, the NH_2_-SiO_2_@RF nanocomposites were prepared by using ethanol/water as a reaction solvent and concentrated ammonia as a catalyst at 30 °C, as shown in Figure 1. At the beginning of the reaction, the silica particles were manufactured through the hydrolysis and condensation of TEOS, around which there were a large number of NH_4_^+^ ions. At the same time, different RF materials were formed during the catalysis of OH^−^ ions. Thus, the shell was formed outside the silica particles due to the electrostatic attraction of NH_4_^+^ and OH^−^ layer by layer. In order to obtain nanocomposites with uniform particle size and spherical morphology, various chemical characteristics (such as the ethanol/water volumetric ratio, the amount of ammonia water, the TEOS dosage, and the resorcinol/formaldehyde molar concentration ratio) were studied. The optimum preparation conditions were an ethanol/water volumetric ratio of 3:1 (total volume 40 mL), an ammonia water volume of 1.25 mL, a TEOS volume of 1.4 mL, and a resorcinol/formaldehyde concentration ratio of 3:1 (0.9 mmol:1.8 mmol). When using the sol–-gel method, this mainly occurs in the reaction between the hydroxyl of RF and the free amino group of APTES in the aqueous solution [24,25].

**Figure 2:**
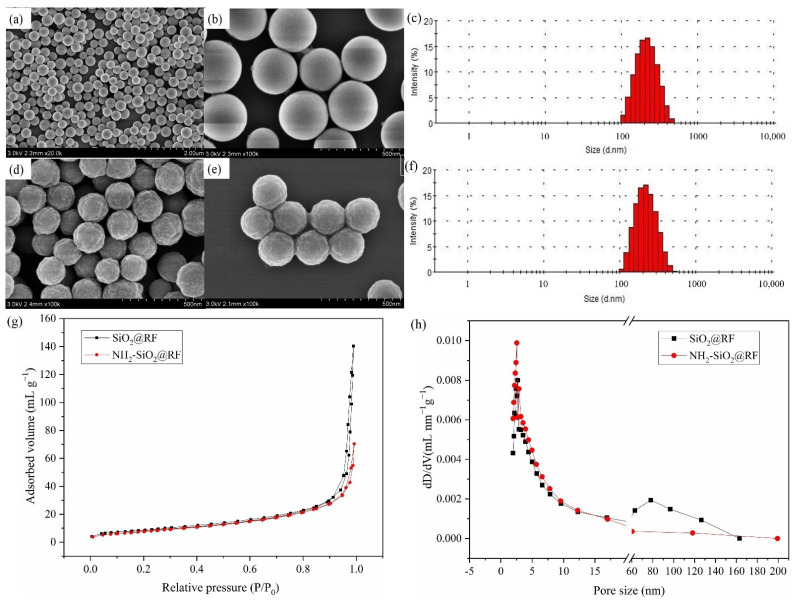
SEM and dynamic light scattering images of SiO_2_@RF (**a**–**c**) and NH_2_-SiO_2_@RF (**d**–**f**). N_2_ adsorption-desorption isotherms (**g**) and pore size distribution (**h**) of SiO_2_@RF and NH_2_-SiO_2_@RF.

The morphological structures and particle size of SiO_2_@RF and NH_2_-SiO_2_@RF were characterized and measured using SEM and dynamic light scattering, and the results are shown in Figure 2. It can be clearly seen that SiO_2_@RF (Figure 2a,b) and NH_2_-SiO_2_@RF (Figure 2d,e) were spherical in terms of morphology, with a relatively rough surface; they had an approximate size of 150–200 nm. The average particle size of the SiO_2_@RF was about 203 nm (Figure 2c), and that of NH_2_-SiO_2_@RF was about 211 nm (Figure 2f).

The surface area and pore size distribution of the nanocomposites were analyzed and tested using BET [26]. The results are given in Figure 2g,h and Table 1. The specific surface area and pore volume of the nanocomposites was reduced from 32.46 m^2^·g^−1^ and 0.217 mL·g^−1^ to 29.42 m^2^·g^−1^ and 0.109 mL·g^−1^, respectively, after SiO_2_@RF amino modification; this is probably because of the introduction of the surface amino group, which occupies part of the pore volume of the SiO_2_@RF material. Figure 2g displays the N_2_ adsorption–desorption isotherm of the material before and after aminoization, exhibiting typical IV isotherms with a sharp capillary condensation step at a high relative pressure (P/P_0_ > 0.9), suggesting the existence of a mesoporous structure [19]. The concentration of pore size distribution and the small average pore size were confirmed by the corresponding BJH pore size distributions (Figure 2h). The BJH pore size of the nanocomposites increased from 2.48 nm to 2.61 nm after SiO_2_@RF amino modification.

**Figure 3:**
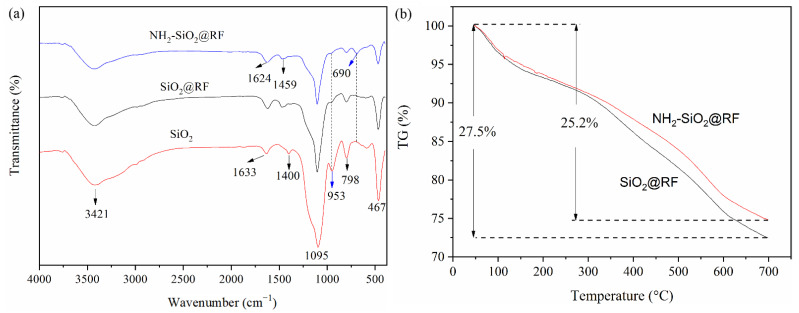
FT-IR spectra of SiO_2_, SiO_2_@RF and NH_2_-SiO_2_@RF (**a**). TGA curves of SiO_2_@RF and NH_2_-SiO_2_@RF (**b**).

FT-IR spectral analysis was carried out on the materials and the results are outlined in Figure 3a. The peaks around 3421 cm^−1^, 1633 cm^−1^, and 1400 cm^−1^ were the stretching vibrations of O-H. The presence of an Si-OH group is confirmed by the peak of O-H bending vibrations at 953 cm^−1^. The peak at 1095 cm^−1^ was assigned to Si-O-Si anti-symmetric stretching vibrations. The peaks at 798 cm^−1^ and 467 cm^−1^ were assigned to Si-O symmetric stretching, and Si-O-Si bending vibrations, respectively [20,27]. For SiO_2_@RF and NH_2_-SiO_2_@RF, the O-H stretching vibration of Si-OH almost disappeared, and the peak at 1624 cm^−1^ represents a C=C stretching vibration of resorcinol on the aromatic ring [12,19]. The peaks at 690 cm^−1^ and 1459 cm^−1^ were assigned to N–H bending vibrations and N–H asymmetric bending vibrations, respectively [28,29]. The FT-IR spectra of NH_2_-SiO_2_@RF showed IR bands at 1469 cm^−1^, which were assigned to the C-H bending vibrations of the alkyl chain of APTES [11,21]. The N–H, C-H of the alkyl chain, and absenting O-H indicated that the RF shell formed on the surface of SiO_2_ and APTES was successfully modified on the surface of SiO_2_@RF.

The thermal stability of SiO_2_@RF and NH_2_-SiO_2_@RF was investigated using thermogravimetric analysis (TGA). The curve of SiO_2_@RF showed that the degradation process occurred between 40 °C and 700 °C and the weight loss was 27.5% (Figure 3b), due to the decomposition of the RF shell on the silica surface [30]. After being modified by APTES, the thermal stability of NH_2_-SiO_2_@RF presumably increased, and the mass loss decreased to 25.2%. It is speculated that this is because the surface amino groups are decomposed before the carbonization of the RF shell on the silica surface.

### 3.2. Effect of NH_2_-SiO_2_@RF Dosage

**Figure 4:**
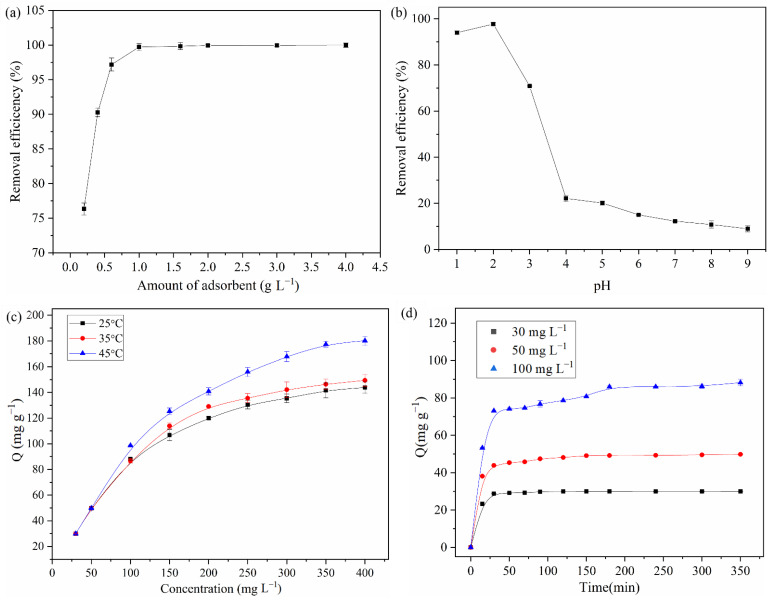
Effects of adsorbent dosage (**a**), solution pH (**b**), initial concentration and temperature (**c**) and contact time (**d**) on adsorption of Cr(VI) onto NH_2_-SiO_2_@RF. Experimental conditions: 150 rpm and Temperature 25 °C. (**a**) C_Cr(VI)_ = 50 mg·L^−1^, 4 h; (**b**) C_NH2-SiO2@RF_ = 1.0 g·L^−1^, C_Cr(VI)_ = 50 mg·L^−1^, 4 h; (**c**) C_NH2-SiO2@RF_ = 1.0 g·L^−1^, pH = 2.0, 24 h (**d**) C_NH2-SiO2@RF_ = 1.0 g·L^−1^, pH = 2.0.

Among the factors influencing adsorption efficiency, the adsorbent dosage is one of the most important. A high dose leads to a high adsorbent cost, which means that the adsorbent cannot be used widely. The adsorption experiment was carried out using the method described above, and the results are shown in Figure 4a. The removal efficiency increased gradually with the increase in the adsorbent amount from 0.2 g·L^−1^ to 1.0 g·L^−1^. As the amount of adsorbent continued to increase from 1.0 g·L^−1^ to 4.0 g·L^−1^, there was almost no further increase in removal efficiency. This is possibly because when the amount of adsorbent used was small, there were fewer active adsorbent sites and they could not completely adsorb Cr(VI) in the solution. With the increase in the amount of adsorbent used, the number of active sites available gradually increased, and the removal efficiency reached the maximum when the active sites of NH_2_-SiO_2_@RF can completely adsorb Cr(VI) in the solution, reaching adsorption equilibrium [6,20,26]. When the amount of the adsorbent continued to increase, the removal efficiency was no longer changed. Therefore, in subsequent experiments, 1.0 g·L^−1^ was selected as the appropriate adsorbent dosage.

### 3.3. Effect of Solution pH 

The solution pH is a key factor affecting the adsorption efficiency of Cr(VI), and the results of adsorption experiments are shown in Figure 4b. As observed, when the solution pH ranged from 1.0 to 2.0, the removal efficiency of Cr(VI) by NH_2_-SiO_2_@RF increased slowly, with the highest removal efficiency (about 98%) found at pH 2.0. Then, the removal efficiency decreased rapidly as the pH increased from 2.0 to 4.0 and continued decreasing to about 9% from pH values of 4.0 to 9.0. The results may be due to the altered morphology of Cr(VI) in solutions at different pH values. Cr(VI) was usually in the form of dichromate (Cr_2_O_7_^2−^) and hydrogen chromate (HCrO_4_^−^) at a pH of 2.0–6.0. When the pH was above 6.0, it was chromate (CrO_4_^2−^), while below 1.0, it was H_2_CrO_4_ [2,12,28]. This phenomenon could likely to be explained by the amino groups on the surface of the material, which were protonated into positively charged −NH_3_^+^ at a low pH, meaning that Cr(VI) can be removed via the electrostatic adsorption of HCrO_4_^−^ and Cr_2_O_7_^2−^ in the solution. The degree of protonation of NH_2_-SiO_2_@RF decreased when the pH of the solution increased, and the number of OH^−^ ions increased in the admixture. There is competition between OH^−^ ions and the Cr(VI) of the anionic morphology for the recognition site of the adsorbent, which results in a decrease in the removal efficiency of Cr(VI). Therefore, the pH was selected as 2.0 in the solution for Cr(VI) removal in subsequent experiments, which is consistent with previous studies [21].

### 3.4. Effect of Initial Concentration and Temperature

The adsorption capacities of NH_2_-SiO_2_@RF increased with the increase in the initial concentration of Cr(VI) and in the temperature, as shown in Figure 4c, which might be because increasing the concentration of Cr(VI) and the temperature was conducive to internal diffusion [1]. Considering the actual usage environment, 25 °C was suitable for practical use. The maximum adsorption capacity for Cr(VI) of NH_2_-SiO_2_@RF was 151.6 mg·g^−1^ at an ambient temperature.

### 3.5. Effect of Contact Time

The adsorption of Cr(VI) (30, 50, and 100 mg·L^−1^) by NH_2_-SiO_2_@RF at different times was investigated at 25 °C to study the adsorption equilibrium time and the dynamic interaction between the adsorbent and adsorbate. As the results presented in Figure 4d show, the adsorption capacity increased rapidly with the increase in time from 0 to 30 min, and it then increased slowly until adsorption equilibrium was reached. The rapid adsorption growth stage could be attributed to the abundant active sites of the adsorbent surface, implying a close connection between NH_2_-SiO_2_@RF and Cr(VI) [13]. Due to the increase in the amount of Cr(VI), the adsorption capacity increased, which implied an increase in the number of collisions between the Cr(VI) anions and the adsorbent. Meanwhile, the adsorption equilibrium time was prolonged with the increase in the Cr(VI) concentration, and it was necessary to set an appropriate contact time.

### 3.6. Adsorption Isotherm Analysis

The Langmuir, Freundlich, Temkin, and Dubinin–Radushkevich (D-R) adsorption isotherm models were used to analyze the test data and attempt to explain the mechanism of NH_2_-SiO_2_@RF for Cr(VI). The Langmuir adsorption isotherm model assumes that the target molecules (or ions) are monolayered, and the defined sites where they are fixed can only hold one molecule layer [31]. The Langmuir isotherm model is shown in Equation (3) and its linear form in Equation (4):

(3) $$q_{e}=\frac{q_{m}K_{L}C_{e}}{1+K_{L}C_{e}}$$

(4) $$\frac{C_{e}}{q_{e}}=\frac{C_{e}}{q_{m}}+\frac{1}{q_{m}KL}$$

where q_m_ (mg·g^−1^) denotes the maximum adsorption capacity, and K_L_ (L·mg^−1^) is the Langmuir constant. R_L_ can describe the basic feature of the Langmuir isotherm model to a certain extent and can be given by Equation (5),

(5) $$RL=\frac{1}{1+KLC_{0}}$$

The value of R_L_ indicates the character of adsorption and the shape of the isotherm; when the adsorption is unfavorable with R_L_ > 1 and favorable with R_L_ < 1, the linear adsorption isotherm becomes R_L_ = 1 [32].

Moreover, the Freundlich isotherm model is an empirical equation based on multilayer adsorption on a heterogeneous surface [33]. Equation (6) and its linear form, given in Equation (7), express this empirical model:

(6) $$q_{e}=K_{f}\cdot C_{e}^{\frac{1}{n}}$$

(7) $$\ln\;q_{e}=\ln\;K_{f}+\frac{1}{n}\ln C_{e}$$

where K_f_ (mg^(1−1/n)^·L^1/n^·g^−1^)and n are Freundlich constants related to adsorption capacity and intensity, respectively. In addition, 1/n ranges between 0 and 1 for a suitable adsorption system.

The Temkin model takes into account the effects of adsorbent–adsorbate interactions. It assumes that the heat of adsorption of all molecules in the layer decreases linearly, rather than logarithmically, with the increasing surface coverage [31]. The Temkin isotherm model is shown in Equation (8) and its linear form is given as Equation (9):

(8) $$q_{e}=\frac{\mathrm{RT}}{b_{T}}\ln\left(K_{T}\cdot C_{e}\right)$$

(9) $$q_{e}=\left(\frac{\mathrm{RT}}{b_{T}}\right)\ln C_{e}+\left(\frac{\mathrm{RT}}{b_{T}}\right)\ln K_{T}$$

where R (8.314 J·mol^−1^·K^−1^) is the ideal gas constant, T (K) is the absolute temperature, K_T_ (L·g^−1^) is the Temkin isotherm equilibrium binding constant, and b_T_ is the Temkin isotherm constant related to the heat of sorption (J·mol^−1^). 

The Dubinin–Radushkevich (D-R) model is a semi-empirical equation in which adsorption follows a pore-filling mechanism with a Gaussian energy distribution onto heterogeneous surfaces. It has been successfully used to quantitatively describe the adsorption of gases onto microporous adsorbents [34]. Equation (10) and its linear form, Equation (11), express this empirical model:

(10) $$q_{e}=q_{m\;}\exp\left(-K_{\mathrm{DR}}\cdot \varepsilon^{2}\right),\;\varepsilon =\mathrm{RTln}\left(1+\frac{1}{C_{e}}\right)$$

(11) $$\ln q_{e}=\ln q_{m}-K_{\mathrm{DR}}{\left[\mathrm{RTln}\left(1+\frac{1}{C_{e}}\right)\right]}^{2}$$

where K_DR_ (mol^2^·kJ^−2^) is the D-R isotherm constant, and ε (kJ·mol^−1^) is the potential Polanyi.

**Figure 5:**
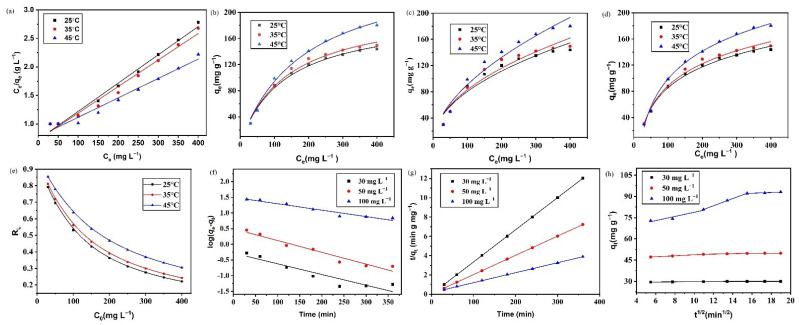
Linear and nonlinear forms of Langmuir isotherm plots (**a**,**b**), nonlinear forms of Freundlich and Temkin isotherm plots (**c**,**d**), separation factor (*R*_L_) at 25 °C, 35 °C and 45 °C (**e**), pseudo-first-order and pseudo-second-order kinetic models (**f**,**g**), intraparticle diffusion model (**h**) on adsorption of Cr(VI) onto NH_2_-SiO_2_@RF. Experimental conditions: (**a**–**e**) C_NH2-SiO2@RF_ = 1.0 g·L^−1^, C_Cr(VI)_ = 50 mg·L^−1^, 150 rpm shaken for 24 h, pH = 2.0; (**f**–**h**) C_NH2-SiO2@RF_ = 1.0 g·L^−1^, 150 rpm shaken for 24 h, Temperature = 25 °C, pH = 2.0.

Both the nonlinear and linearized forms of the four models were used to obtain best fits to the adsorption equilibrium data. The obtained curves are illustrated in Figure 5a–c. Table 2 summarizes the isotherm parameters and correlation coefficients obtained from the best fits to data using the nonlinear and linearized forms of the four models, respectively. The Langmuir and Temkin models fitted the adsorption process reasonably well, followed by the Freundlich model. The maximum adsorption capacity calculated by the Langmuir model was closer to the experimental results. According to the assumption of the Langmuir adsorption isothermal model, it could be concluded that the adsorption of Cr(VI) in the solution by the NH_2_-SiO_2_@RF composite likely constituted monolayer adsorption. Meanwhile, the calculated 1/n values of the Freundlich model were smaller than 1, indicating that the adsorption reaction could easily occur. Furthermore, the values of *R*_L_ were much smaller than 1 at all the three temperatures, which indicated that the adsorption of Cr(VI) on NH_2_-SiO_2_@RF was advantageous. 

### 3.7. Adsorption Kinetic Model 

Adsorption kinetic models not only describe the rate of adsorption but can also be helpful for understanding the mechanism of adsorption. The pseudo-first-order and pseudo-second-order models were used to analyze the data [15]. The quasi-first-order kinetic model can be descripted by Equation (12):

(12) $$\log\left(q_{e}-q_{t}\right)=logq_{e}-\frac{k_{1}t}{2.303}$$

where q_t_ (mg·g^−1^) is the adsorption capacity at time t, and k_1_/min^−1^ is the quasi-first-order rate constant.

The quasi-second-order model can be given in the form of Equation (13):

(13) $$\frac{t}{q_{t}}=\frac{1}{k_{2}\cdot {q_{e}}^{2}}+\frac{1}{q_{e}}t$$

where k_2_ ((g·mg^−1^)·min^−1^) is the rate constant of quasi-second-order adsorption and the values of the constants are calculated from the straight-line plots of t/q_t_ versus t.

The simulation results are illustrated in Figure 5f,g and Table 3. The size of the obtained linear R^2^ was calculated using two kinetic equations to select the best matching model. The results showed that the R^2^ values calculated from the pseudo-second-order equation were larger than those obtained from the pseudo-first-order equation. The value of the equilibrium adsorption capacity Qcal calculated from the pseudo-second order equation was similar to the experimental value Qexp. Meanwhile, the value of k_2_ decreased with the increase in the Cr(VI) concentration in the solution. In short, the results suggested that the pseudo-second-order model was suitable for the description of the adsorption kinetics and that chemisorption played an important role in the adsorption process [35]. 

### 3.8. Intraparticle Diffusion Model

The intraparticle diffusion model [36], the Weber–Morris plot (q_t_ versus t^1/2^), is often used to measure the functional relationship in the adsorption process, which can be calculated using Equation (14):

(14) $$q_{t}=k_{i}t^{1/2}+C$$

where k_i_ (mg·g^−1^·min^−1/2^) is the diffusion rate constant, and the intercept C relates to the thickness of the boundary layer.

The plots of q_t_ versus t^1/2^ for Cr(VI) adsorption by the adsorbent, which were linearly fitted, are displayed in Figure 5h.

The fitting yields a multi-linear model composed of three parts, indicating that the adsorption process consists of three steps. The first section, the process of external diffusion, indicates that the Cr(VI) in the solution passed through the film or the boundary layer via molecular diffusion or convection diffusion into the outer surface of NH_2_-SiO_2_@RF. The second step is the internal diffusion process, and the model parameters were generally calculated using this section when Cr(VI) is transferred to the inner surface of NH_2_-SiO_2_@RF through pore diffusion. The third step is relatively simple, representing the adsorption equilibrium stage [37]. As shown in Figure 5h, none of the linear straight lines passed through the origin, indicating that intraparticle diffusion was not the only a rate-limiting step, but that there were other adsorption stages. Moreover, the intraparticle diffusion rate constant *k*_i_. increased with the increase in the Cr(VI) concentration. This result likely occurred because the diffusion driving force increased with the increase in the Cr(VI) concentration, which accelerated the rate of Cr(VI) diffusion into the material surface through the hole in the solution. The intercepting C reflects the influence of the boundary layer, and the large value implied that the boundary layer effect was also large [26].

### 3.9. Thermodynamic Study

In order to explore the adsorption process further, a thermodynamic study was conducted at 298, 308, and 318 K. thermodynamic parameters [26]. Gibbs free energy ΔG, standard entropy ΔS, and standard enthalpy changes ΔH associated with the adsorption of Cr(VI) onto NH_2_-SiO_2_@RF can be calculated from the temperature-dependent adsorption isotherm using the following equations:

(15) $$∆G=-\mathrm{RT}\;\ln K_{d}$$

(16) $$\ln K_{d}=-\frac{∆H}{\mathrm{RT}}+\frac{∆S}{R}$$

(17) $$K_{d}=\frac{q_{e}}{C_{e}}$$

where K_d_ is the solid–liquid distribution coefficient. 

ΔS and ΔH are calculated from the intercept and slope of the linear plot of lnK_d_ vs. 1/T (lnK_d_ = −2.418/T + 3.9589 correlation coefficient: 0.9923), respectively. The calculated values are tabulated in Table 4. The negative ΔG values (−9.79~−10.45 kJ·mol^−1^) illustrate the feasibility and spontaneity of the adsorption process [6,36]. The positive ΔH value (0.02 kJ·mol^−1^) indicates that the adsorption process was endothermic. The positive value of ΔS (32.91J·mol^−1^·K^−1^) demonstrated the increase in randomness at the solid–liquid interface during Cr(VI) adsorption onto NH_2_-SiO_2_@RF [23,37].

### 3.10. Anti-Interference Examinations

**Figure 6:**
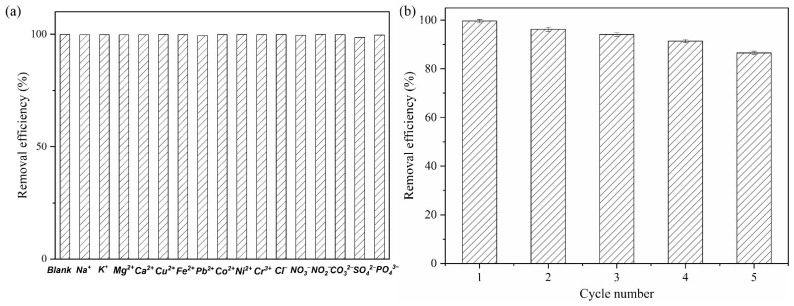
Effect of coexisting ions on adsorption of Cr(VI) onto NH_2_-SiO_2_@RF (**a**). Removal efficiency of NH_2_-SiO_2_@RF by five adsorption-desorption cycles of regeneration (**b**). C _familiar coexisting ions_ = 300 mg·L^−1^, C_NH2-SiO2@RF_ = 1.0 g·L^−1^, C_Cr(VI)_ = 30 mg·L^−1^, 150 rpm shaken for 24 h, Temperature = 25 °C, pH = 2.0.

To evaluate the potential of the adsorbent in real water samples, the effect of ions that may coexist with Cr(VI) in a solution on their adsorption efficiency was investigated. The familiar coexisting ions (K^+^, Na^+^, Mg^2+^, Ca^2+^, Fe^2+^, Cu^2+^, Pb^2+^, Co^2+^, Ni^2+^, Cr^3+^, Cl^−^, NO_3_^−^, NO_2_^−^, CO_3_^2−^, SO_4_^2−^ and PO_4_^3−^) were studied. In brief, 300 mg·L^−1^ of the above ions with 5 mL 30 mg·L^−1^ Cr(VI) solution was used for the above adsorption experiment. As the results in Figure 6a show, the influence of the concentration of cations and anions, which was studied ten times, had hardly any effect. This indicates that there was almost no competition between Cr(VI) and the aforementioned ions, which would make the recovery of the adsorbent from wastewater samples advantageous.

### 3.11. Desorption and Reusability

The reusability of the material is a key factor in evaluating whether the adsorbent has potential practical application value. A lower pH was beneficial for adsorption, but conversely, alkaline conditions could be helpful for the desorption of Cr(VI). The adsorption–desorption experiment of the above-mentioned parts, with 0.5 mol·L^−1^ NaOH as the desorbent, was used to investigate the reusability times of NH_2_-SiO_2_@RF. As the results in Figure 6b show, the removal efficiency of 30 mg·L^−1^ Cr(VI) is still maintained at more than 85% after five adsorption–desorption cycles of regeneration, which indicated that the material has good reusability and high stability for Cr(VI) adsorption in solution.

### 3.12. Comparison of Adsorption Properties

Table 5 presents a comparison of the adsorption capacities of NH_2_-SiO_2_@RF with other amino-functionalized adsorbents for Cr(VI) removal. The maximum adsorption capacity based on the Langmuir model (*q_m_*) was 272.6 mg·g^−1^, which was higher than those of amine-functionalized mesoporous silicas reported in the literature (Table 5), excluding the Cr(VI) adsorption capacity (330.9 mg·g^−1^) of Kim et al. [22]. 

### 3.13. Primary Stage of Practical Application

**Figure 7:**
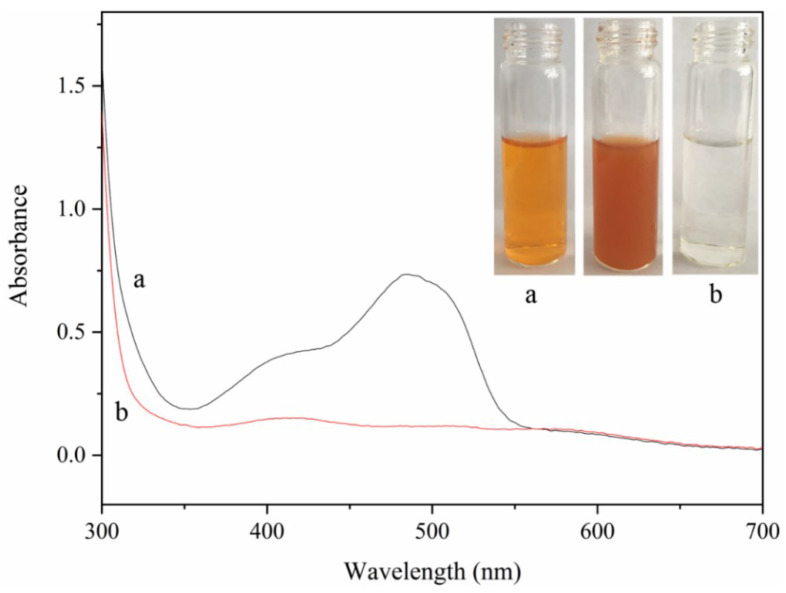
The Uv-vis spectrum and photos of tannery wastewater before (**a**) and after (**b**) adsorption by NH_2_-SiO_2_@RF. Experimental conditions: V_wastewater_ = 5 mL, C_NH2-SiO2@RF_ = 1.0 g·L^−1^, 150 rpm shaken for 24 h, Temperature = 25 °C, pH = 2.0.

Tannery wastewater was selected as the actual water sample to further verify the practical application of the materials. The tannery wastewater was filtered using a hydrophilic PTFE syringe filter (0.22 μm). After that, the Cr(IV) in tannery wastewater was treated under the optimum conditions selected by the batch adsorption studies. After the adsorption procedures, the tubes containing the mixture were centrifuged at 7500 rpm for 10 min. Then, the supernatant liquors were acidized using phosphoric acid and complexed with DPC; they were detected with full wavelength scanning using a UV-vis spectrophotometer. Most of the yellow color came from tannin extracts and other pigments in the wastewater. Impurities had little effect on chromium detection and were removed via filtration. The results obtained after the adsorption experiment are illustrated in Figure 7, which shows that the initial color of the sample was light brown-yellow; the color deepened after ultrasonic dispersion with the addition of the adsorbent, and, finally, the solution became clear and transparent after the adsorption reaction (Figure 7a,b). On the corresponding Uv spectrum, there were two obvious valley peaks in the initial tannery wastewater. After material adsorption, the valley peak disappeared and was basically close to the baseline. Owing to the multiple effects of the functional groups and structures of the material, complex compounds in tannery wastewater can be effectively removed, showing the material’s potential practical application value.

## 4. Conclusions

In summary, a nanocomposite adsorbent with a high adsorption capacity, NH_2_-SiO_2_@RF, was prepared using the Stöber method and amination onto the surface via the sol–gel method. The nanocomposite adsorbent NH_2_-SiO_2_@RF was successfully used for the removal of Cr(VI) from tannery wastewater. The materials showed homogeneous morphology and high thermostability. Only a small amount of NH_2_-SiO_2_@RF nanocomposites (1.0 g·L^−1^) was needed to achieve the effective removal of Cr(VI) at a low pH of 2.0, and the selectivity was good (i.e., the other coexisting ions were almost undisturbed). The adsorption behaviors of Cr(VI) were in accordance with the Langmuir and Temkin isotherm models and pseudo-second-order kinetic models, and intraparticle diffusion was not the only rate-limiting step. The absorbent’s high adsorption capacity, excellent anti-ion interference, and reuse performance demonstrated its potential practical application value. Notably, NH_2_-SiO_2_@RF was successfully preliminarily applied to achieve the adsorption and removal of Cr(VI) in tannery wastewater. Given the combined advantages of the NH_2_-SiO_2_@RF nanocomposites, we can deduce that this material has significant potential for the removal of Cr(VI) from industrial wastewater and environmental water.

## Figures and Tables

**Table 1:** BET surface areas, pore volumes and pore size of NH_2_-SiO_2_@RF.

Nanocomposite	Surface Area/m^2^·g^−1^	Pore Volumes/mL·g^−1^	BJH Pore Volumes/nm
SiO_2_@RF	32.46	0.217	2.61
NH_2_-SiO_2_@RF	29.42	0.109	2.48

**Table 2:** Adsorption parameters obtained from the Langmuir, Freundlich, Temkin, and Dubinin-Radushkevich (D-R) isotherms on adsorption of Cr(VI) onto NH_2_-SiO_2_@RF.

Models	T/K	Linear			Nonlinear		
Langmuir		q_m_/mg·g^−1^	K_L_/L·mg^−1^	R^2^	q_m_/mg·g^−1^	K_L_/L·mg^−1^	R^2^
	298	189.6	0.0088	0.9887	196.4	0.0075	0.9918
	308	198	0.0078	0.9822	208.2	0.0072	0.9864
	318	212.2	0.0057	0.9727	272.6	0.0053	0.9919
Freundlich		1/n	K_f_/mg^(1−1/n)^·L^1/n^·g^−1^	R^2^	1/n	K_f_/mg^(1−1/n)^·L^1/n^·g^−1^	R^2^
	298	0.599	4.735	0.9330	0.473	9.075	0.948
	308	0.587	4.973	0.9230	0.483	8.971	0.9301
	318	0.59	5.063	0.8860	0.547	7.301	0.9552
Temkin		b_T_/J·mol^−1^	K_T_/L·g^−1^	R^2^	b_T_/J·mol^−1^	K_T_/L·g^−1^	R^2^
	298	54.13	0.063	0.9914	54.15	0.065	0.9917
	308	52.48	0.060	0.9860	52.58	0.061	0.9862
	318	43.27	0.051	0.9919	43.28	0.052	0.9921
D-R		q_m_/mg·g^−1^	K_DR_/mol^2^·J^−2^	R^2^	q_m_/mg·g^−1^	K_DR_/mol^2^·J^−2^	R^2^
	298	126.5	236.7	0.8821	134.4	424.4	0.9002
	308	129.0	228.6	0.8736	141.6	444.7	0.9055
	318	148.4	237.1	0.8556	169.5	584.6	0.9012

**Table 3:** Adsorption parameters obtained from the kinetic equation and intraparticle diffusion model for the adsorption of Cr(VI) by NH_2_-SiO_2_@RF at different concentrations.

C /mg·L^−1^	Q_exp_ /mg·g^−1^	Pseudo-First-Order			Pseudo-Second-Order			Intraparticle Diffusion		
		Q_cal_ /mg·g^−1^	K_1 _ /min^−1^	R^2^	Q_cal _ /mg·g^−1^	K_2_ /g·mg^−1^·min^−1^	R^2^	k_i_ /mg·g^−1^·min^−1/2^	C	R^2^
30	30.0	0.52	0.0079	0.8751	30.01	0.048	1	0.031	29.48	0.9781
50	49.8	3.13	0.0087	0.9500	50.15	0.0080	1	0.142	47.52	0.9964
100	93.1	31.5	0.0047	0.9457	97.09	6.1 × 10^−4^	0.9998	2.538	52.94	0.9995

**Table 4:** Thermodynamics parameter of Cr(VI) removal onto NH_2_-SiO_2_@RF.

T/K	ΔG/kJ·mol^−1^	ΔH/kJ·mol^−1^	ΔS/J·mol^−1^·K^−1^
298	−9.788	0.02008	32.91
308	−10.12		
318	−10.45		

**Table 5:** Comparison of adsorption capacities with similar materials.

Adsorbent	Amine Functionalization	Adsorption Capacity/mg·g^−1^	Reference
NH_2_-SiO_2_@RF	APTES	272.6	this study
NH_2_-MCM-41/PMNCs	APTES	20	[23]
NH_2_-MCM-41	APTES	86.4	[38]
NH_2_-PNIPAm	APTES	123.8	[39]
NH_2_-pSiO_2_	APTES	50	[40]
NH_2_-MSNs	APTES	42.2	[21]
NH_2_-MPS	APTMS	83.5	[41]
NH_2_-SB_A_-15	DAEAPTS	330.9	[22]
NH_2_-MPHC	HDA	142.9	[42]

Abbreviation: APTES = 3aminopropyltriethoxysilane; APTMS = 3-aminopropyltrimethoxysilane; DAEAPTS = N-(2-Aminoethyl-3-aminopropyl) trimethoxysilane; HDA = Hexadecylamine.

## Data Availability

All data included in this study are available upon request by contacting the corresponding author.
